# Safety and immunogenicity of Vi-diphtheria toxoid typhoid conjugate vaccine among children below 2 years: a systematic review and meta-analysis

**DOI:** 10.3389/fmicb.2024.1385834

**Published:** 2024-04-05

**Authors:** Amira Mohamed Taha, Khaled Abouelmagd, Abdelrahman Mohamed Mahmoud, Mohamed Hamouda Elkasaby, Dang Nguyen, Ryan Ahmed, Pari Patel, D. Katterine Bonilla-Aldana, Camila Luna, Alfonso J. Rodriguez-Morales

**Affiliations:** ^1^Faculty of Medicine, Fayoum University, Fayoum, Egypt; ^2^Cardiology Department, Faculty of Medicine, Al-Azhar University, New Damietta, Egypt; ^3^Faculty of Medicine, Menoufia University, Menoufia, Egypt; ^4^Faculty of Medicine, Al-Azhar University, Cairo, Egypt; ^5^Massachusetts General Hospital, Corrigan Minehan Heart Center, Harvard Medical School, Boston, MA, United States; ^6^Department of Biological and Chemical Sciences, New York Institute of Technology, New York, NY, United States; ^7^Research Unit, Universidad Continental, Huancayo, Peru; ^8^Faculty of Health Sciences, Universidad Científica del Sur, Lima, Peru; ^9^Grupo de Investigación Biomedicina, Faculty of Medicine, Fundación Universitaria Autónoma de las Américas-Institución Universitaria Visión de las Américas, Pereira, Colombia; ^10^Gilbert and Rose-Marie Chagoury School of Medicine, Lebanese American University, Beirut, Lebanon

**Keywords:** typhoid fever, vaccination, safety, efficacy, systematic review

## Abstract

**Background:**

The Vi-diphtheria toxoid typhoid conjugate vaccine (Vi-DT) has shown promising results in preventing typhoid fever in children under 2 years of age. However, a thorough assessment of its safety and immunogenicity is required to inform vaccination strategies. This systematic review and meta-analysis aimed to determine the safety and immunogenicity of Vi-DT in children below 2 years.

**Methods:**

We systematically searched multiple databases, including PubMed, Web of Science, and Scopus, for relevant studies published up to September 2023. We included studies reporting on the safety and immunogenicity outcomes of Vi-DT compared to the control or Vi-tetanus toxoid conjugated vaccine (Vi-TT) in children below 2 years. We applied a random-effects model for meta-analysis using RevMan 5.4. We expressed the results as risk ratio (RR) with a 95% confidence interval (95%CI).

**Results:**

In this analysis, five studies were selected, encompassing 1,292 children under 2 years who received the Vi-DT vaccine. No significant difference in immediate reactions was observed within 30 min post-vaccination between Vi-DT and control groups (RR: 0.99 [95% CI: 0.19, 5.26]), nor between Vi-DT and Vi-TT groups. For solicited adverse events within 4 weeks, the VI-DT group showed no significant increase in adverse events compared to control (RR: 0.93 [95% CI: 0.78, 1.12]) or Vi-TT (RR: 0.86 [95% CI: 0.69, 1.07]). Similarly, within 7 days post-vaccination, risk ratios indicated no significant differences in adverse events between the groups. The 4-week seroconversion rate was significantly higher in the Vi-DT group compared to the control (RR: 1.99 [95% CI: 1.07, 3.69]), but no difference was found between Vi-DT and Vi-TT. Adverse events associated with typhoid conjugate vaccines were predominantly non-serious, including fever and injection site reactions. Serious adverse events were rare but included conditions like pneumonia and gastroenteritis.

**Conclusion:**

This meta-analysis highlights Vi-DT safety and immunogenicity in six to 24-month-old children. The findings support the use of this Vi-DT to expand typhoid vaccination in endemic regions, in line with WHO’s strategy.

## 1 Introduction

Typhoid fever arises due to an intestinal infection instigated by the Gram-negative pathogen *Salmonella enterica* serovar Typhi, commonly referred to as *S. typhi*. The primary route of human infection by this bacterium is through ingesting food and water that have been contaminated (Brooks et al., 2005). The progression of an infection caused by *S. typhi* typically follows a sequential pattern. Initially, patients experience a fever in the first week, which intensifies during the second week. The manifestation of symptoms such as abdominal discomfort, constipation, and the emergence of maculopapular rashes also characterizes this period. As the infection advances into the third week, patients may experience more severe complications, including hepatosplenomegaly, ileocecal perforation, peritonitis, and septic shock. In the absence of appropriate medical intervention, typhoid fever has the potential to lead to mortality (Saha et al., 2019).

*S. typhi* predominantly impacts areas where clean water and sanitation facilities are scarce, posing a substantial public health challenge in numerous regions across the globe, particularly in less developed countries (Brooks et al., 2005; Saha et al., 2019). The World Health Organization (WHO) estimated in 2019, every year, approximately 9 million people are affected by typhoid fever causing around 110,000 deaths (Typhoid, 2023). The latest estimates reveal an increase in this condition; Roughly 12 million cases of typhoid occur each year, leading to more than 128,000 deaths annually. The highest incidence of *S. typhi* is in Asia and Africa, where rates exceed 100 per 100,000 person-years, and children are the most affected (Crump et al., 2004; Ochiai et al., 2008; Jamka et al., 2019).

While typhoid fever can be effectively treated with antibiotics, the increasing issue of antimicrobial drug resistance and the associated high expenses present significant challenges, particularly in regions with lower and moderate-income levels (LMICs) where the disease is prevalent (Jeon et al., 2018; Syed et al., 2020). Prevention of *S. typhi* focuses on better antibiotic management, public sanitation, clean water access, safe food handling, and hygiene education. According to the WHO, Vaccination is the mainstay and one of the most cost-effective measures (World Health Organization, 2019).

Several vaccines have been developed to prevent *S. typhi* infection. They are divided into three categories: live attenuated whole-cell Ty21a vaccine, purified unconjugated Vi polysaccharide vaccine (ViPS), and Vi conjugated vaccines (TCVs). The FDA has approved two vaccine categories, Ty21a and ViPS, for use in the USA (Hohmann et al., 1996; Crump and Mintz, 2010).

The Ty21a vaccine is an orally administered live attenuated vaccine that requires four doses. It is recognized for its ability to trigger a localized immune response in the intestinal tract by stimulating the production of lipopolysaccharides (Mazzone et al., 1994; Amicizia et al., 2017). TCVs have been proven useful in giving long-term immunity. A study of children in Nepal found that a single TCV treatment provided long-term protection. Specifically, 2 years after immunization, the vaccine’s efficacy in preventing blood culture-positive typhoid fever was similar with previous interim efficacy data, with an efficacy of 81.6% (Shakya et al., 2021). Furthermore, the study observed no significant difference in vaccine efficacy between the first 12 months and the subsequent period (Shakya et al., 2021).

Despite being effective, there are certain limitations to the use of these vaccines in countries where typhoid is endemic and in children under the age of two. The Vi-PS and Ty21a vaccines provide similar levels of protection within 2 years of vaccination. However, neither is authorized for use in infants particularly vulnerable to typhoid. Their protection is not sustained for over a few years (Syed et al., 2020). Additionally, Ty21a is exclusively accessible in capsule form and is not suitable for children under the age of five. It necessitates multiple doses for efficacy and, being a live organism, is not advisable for individuals with compromised immune systems or during pregnancy. Moreover, it demands strict cold chain maintenance, a significant constraint in resource-limited areas (World Health Organization, 2019).

Conjugating a carrier protein to the polysaccharide has effectively developed vaccines against Pneumococcal, Meningococcal, and *Haemophilus influenzae* infections as well as the Typhoid ViPS vaccine (Medise et al., 2019). The polysaccharide’s antigenic characteristics transform into a T cell-dependent antigen by attaching to a carrier protein. This alteration leads to an improved memory response and the development of protective immunity in both children and adults (World Health Organization, 2019).

In 2017, the World Health Organization (WHO) accorded prequalification status to Bharat Biotech’s Vi-TT, the inaugural Typhoid Conjugate Vaccine (TCV) developed in India. This vaccine received its initial license from the Drugs Controller General of India (DCGI) in 2013, permitting its use in the private market for children 6 months and older (Mohan et al., 2015; Burki, 2018). A second TCV conjugated to a nontoxic mutant of diphtheria toxoid (Vi-CRM_197_) (TYPHIBEV, Biological E, India) has been licensed by the DCGI and prequalified by the WHO in 2020 (VaccinesWork, 2023).

Typhoid fever remains a public health problem in multiple countries, requiring integrative efforts to control the disease. Enteric fever, including typhoid and paratyphoid fevers, remains a significant public health concern in many parts of the world, particularly in areas with poor sanitation and limited access to clean water (Syed et al., 2020). Efforts to prevent and treat enteric fever have included vaccination programs, improvements in sanitation infrastructure, and public health education. However, challenges persist, and the disease burden in certain regions is still high (Syed et al., 2020).

The Vi-diphtheria toxoid typhoid conjugate vaccine (Vi-DT) represents a promising advancement in typhoid prevention, which combines the antigenic ViPS from *S. typhi* with the immunogenic diphtheria toxoid protein (Masuet-Aumatell and Atouguia, 2021). Various studies have suggested the safety and efficacy of Vi-DT and other typhoid vaccines in individuals aged 2 years and above (Levine et al., 1987; Kossaczka et al., 1999; Medise et al., 2019). Nonetheless, there is limited available data on the safety and effectiveness of Vi-DT in children under the age of two. The objective of this meta-analysis is to offer a thorough evaluation of Vi-DT’s safety and efficacy in this specific age group.

## 2 Methods

This review is registered at PROSPERO CRD42023471203^1^ and reported in compliance with PRISMA criteria (Moher et al., 2009).

### 2.1 Search strategy and eligibility criteria

Up to September 2023, PubMed, Web Sciences, and Scopus were searched with a constraint on papers written in English and reporting the results of the Vi diphtheria toxoid vaccine (Vi-DT) in children under 2 years of age.

We incorporated all studies that provided the information necessary to assess the vaccine’s efficacy and safety in children under two.

After removing duplicate articles, two researchers assessed the eligibility of the remaining retrieved articles by initially reviewing the titles and abstracts before proceeding to a full-text evaluation. Differences between the two investigators were resolved through discussion and mutual agreement.

### 2.2 Data extraction and quality assessment

In each study, we took the first author’s name, the publication year, the nation, the study population’s characteristics (sample size, percentage of men, age distribution, administration mode, and volume injection), anti-Vi IgG geometric mean titers (GMT), seroconversion, and adverse events (AEs) out. Two authors conducted an assessment of bias in the included studies utilizing the Risk of Bias Tool 2 (ROB-2) (The Cochrane Collaboration, 2023), which considers factors such as the randomization method, deviations from the intended interventions, missing outcome data, assessment of the outcome, selection of the reported results, and the overall risk of bias.

### 2.3 Data synthesis

RevMan^®^ 5.4 for Windows was used for the statistical analysis. If the *p*-value was below 0.05, the results were interpreted as significant for dichotomous outcomes into risk ratios (RR) with their respective 95% confidence intervals (95%CI). The heterogeneity between the studies was evaluated using Cochran’s Q test, where significant heterogeneity is defined as *I*^2^ > 50%. We performed subgroup analyses according to the comparison group, whether it’s another typhoid vaccine or control group, including placebo or any other non-typhoid vaccine.

## 3 Results

### 3.1 Study selection

**Figure 1:**
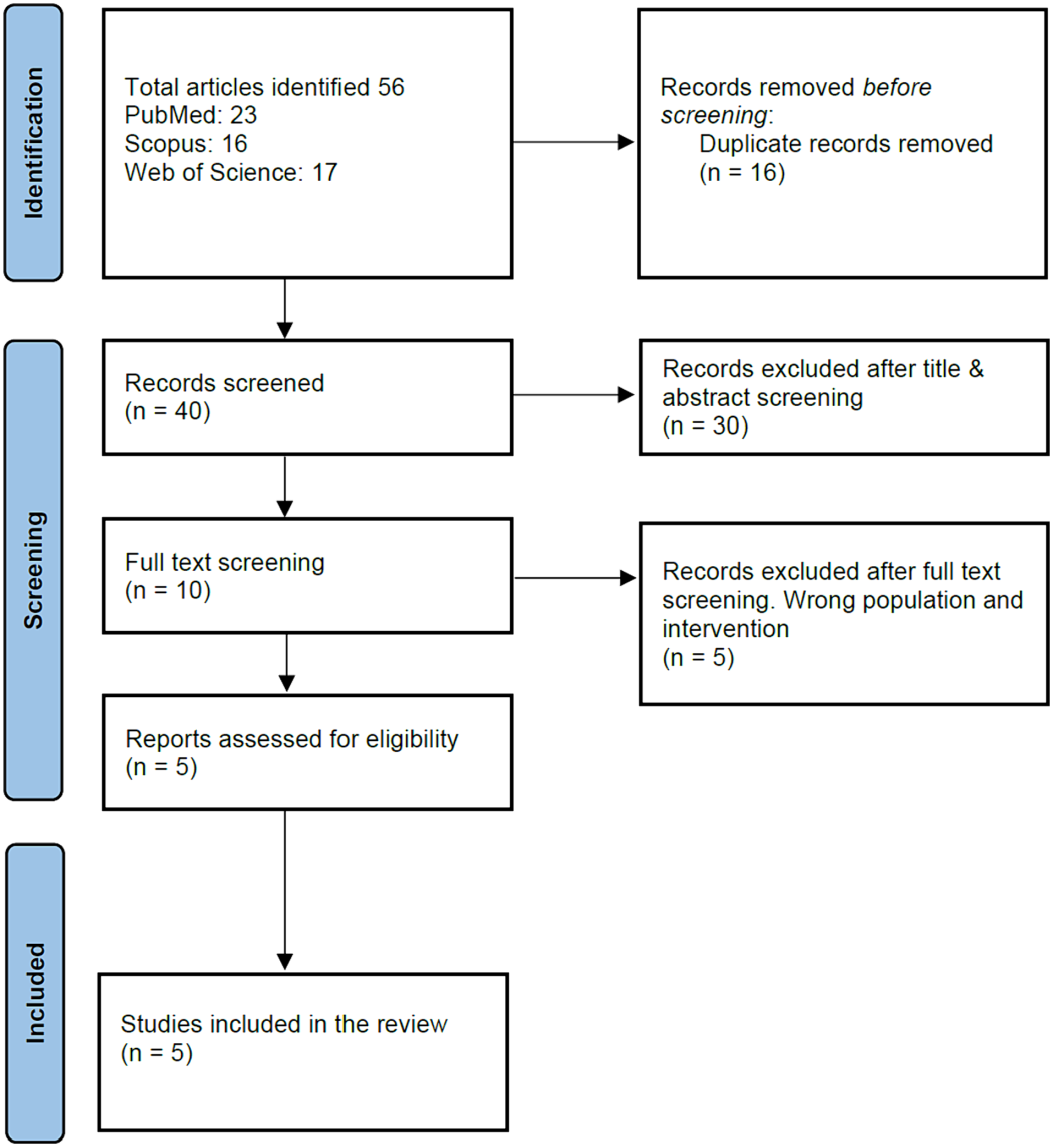
PRISMA diagram.

From the extensive search across databases like PubMed, Web of Science, and SCOPUS up to September 2023, we identified 56 articles. Duplicate articles, amounting to 16, were systematically discarded. A preliminary screening based on titles and abstracts resulted in the removal of another 30 articles, narrowing down our list further. An exhaustive full-text review of the remaining articles was undertaken. Among these, five articles were excluded because they did not meet our strict inclusion criteria, which primarily centered on investigating the safety and immunogenicity of Vi-DT in children under 2 years of age. Ultimately, we arrived at a concise list of five articles in our meta-analysis and systematic review. The PRISMA flow chart comprehensively outlines the detailed selection process, depicted in Figure 1.

All five chosen studies (Capeding et al., 2020; Medise et al., 2020; Carlos et al., 2022; Kumar Rai et al., 2022; Chaudhary et al., 2023) were randomized controlled trials. Geographically, two studies originated from Nepal, with the others hailing from the Philippines, Indonesia, and Korea. The studies encompassed a sample size of 1,292 individuals, of which 826 (63.93%) were male. The age group targeted across these studies predominantly ranged from 11 to 17 months; however, one study did not specify this detail (Chaudhary et al., 2023). The principal mode of administration across the studies was intramuscular injection, utilizing a volume of 0.5 mL. There was a marked variation in administering the second dose of the Vi-DT typhoid conjugate vaccine in the study conducted by Capeding et al., wherein a volume of 0.25 mL was utilized. The studies under review compared the single dose (SD) or multiple doses (MD) of Vi-DT typhoid conjugate vaccine against several controls: meningococcal conjugate vaccine (Carlos et al., 2022), Typbar-Typhoid Conjugate Vaccine (Vi-TT) (Kumar Rai et al., 2022; Chaudhary et al., 2023), inactivated polio vaccine (IPV) (Medise et al., 2020), and placebo such as sodium chloride (Capeding et al., 2020), the baseline characteristics of included studies demonstrated in Table 1.

**Table 1:** Summary of included studies.

ID	Group	Country	Sample size	Age, mean	Sex (Male), n	Administration route	Volume injected
Capeding 2020	Vi-DT typhoid conjugate vaccine (first dose)	Korea	114	11.4	57	IM	0.5 mL
	Vi-DT typhoid conjugate vaccine (second dose)		114	11.7	52		0.25 mL
	0.5 mL of 0.9% sodium chloride		57	11.35	29		0.5 mL
Carlos 2022	Vi-DT typhoid conjugate vaccine (first dose)	Philippines	155	14	84	IM	0.5 mL
	Vi-DT typhoid conjugate vaccine (second dose)		155	12.4	78		
	meningococcal conjugate vaccine		62	12.6	33		0.5 mL
Chaudhary 2023	Vi-DT typhoid conjugate vaccine (first dose)	Nepal	78	–	155	IM	0.5 mL
	Vi-TT typhoid conjugate vaccine (first dose)		26	–	52		
Medise 2020	Vi-DT typhoid conjugate vaccine (first dose)	Indonesia	100	14.14	53	IM	0.5 mL
	Inactivated polio vaccine (IPV)		100	14.73	50		0.5 mL
Rai 2022	Vi-DT typhoid conjugate vaccine (first dose)	Nepal	450	14.1	232	IM	0.5 mL
	Vi-TT typhoid conjugate vaccine (first dose)		150	14.1	81		

Vi-DT, diphtheria toxoid conjugated Vi-polysaccharide vaccine; Vi-TT, Vi-tetanus toxoid conjugated vaccine; IM, intramuscular.

### 3.2 Quality assessment

**Figure 2:**
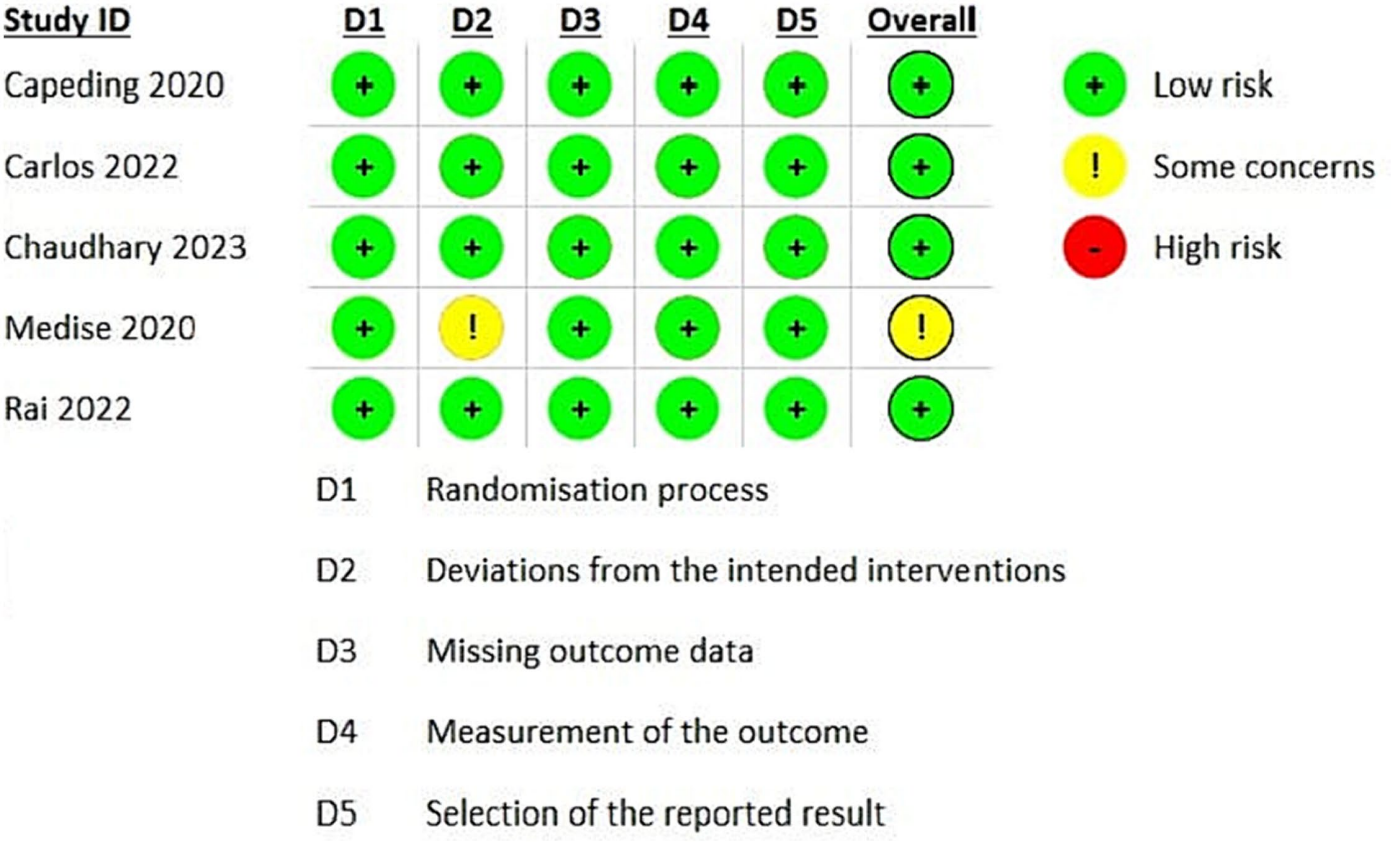
Risk of bias assessment.

Using the Cochrane ROB2 tool, most studies exhibit a low risk of bias in most domains, indicating a robust and reliable methodology. However, there are some concerns regarding the Medise 2020 study (Medise et al., 2020), necessitating a cautious interpretation of these findings (Figure 2).

### 3.3 Outcomes

#### 3.3.1 Safety outcomes

##### 3.3.1.1 Immediate reactions within 30 min post-vaccination

**Figure 3:**
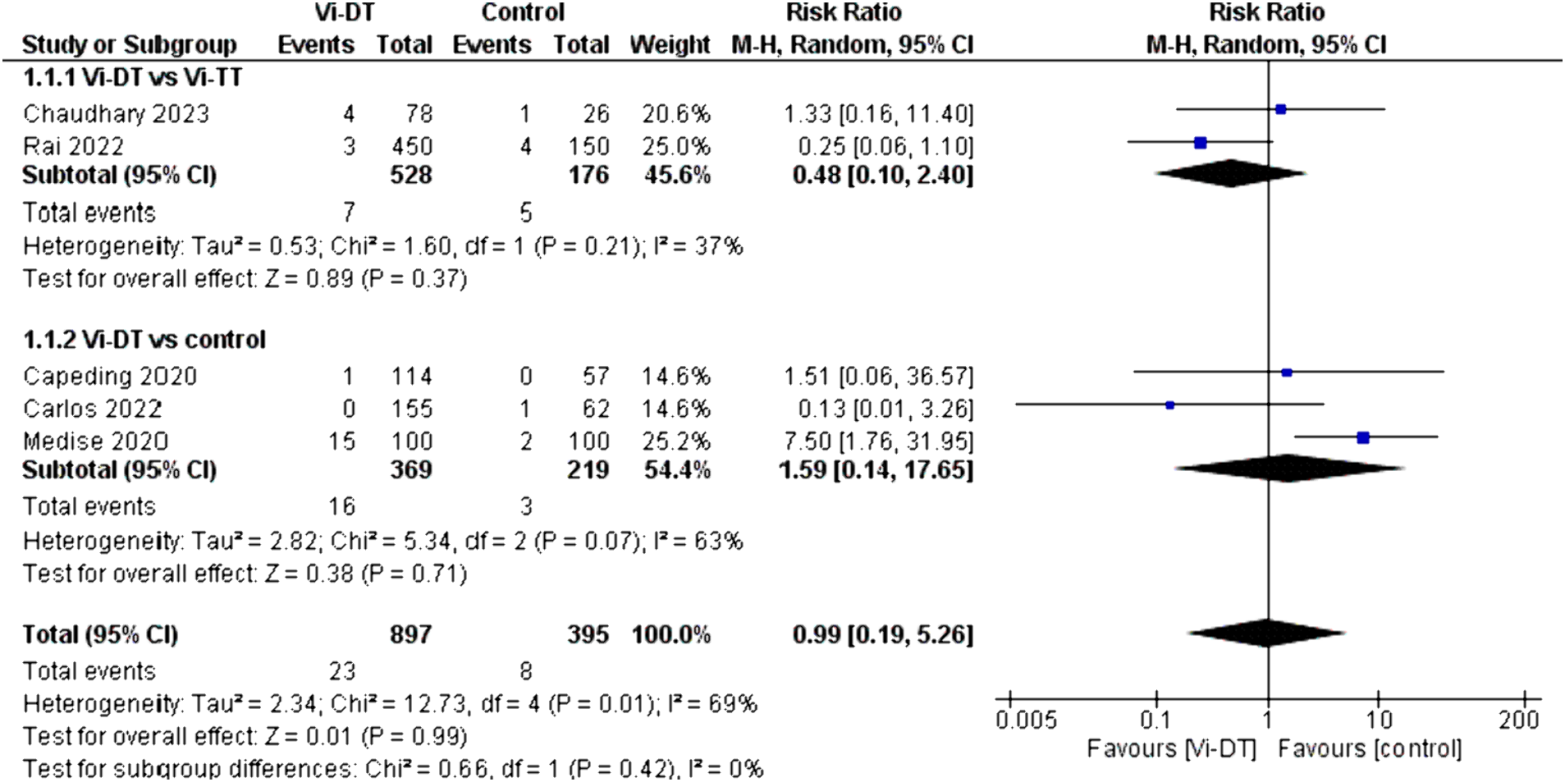
Immediate reactions within 30 min post-vaccination.

Overall, the combined analysis did not show a statistically significant difference in the immediate reactions within 30 min post-vaccination between the Vi-DT group and the control, with a risk ratio of 0.99 [95% CI: 0.19, 5.26], suggesting that Vi-DT does not increase the immediate reaction rate compared to control (Figure 3). This analysis had considerable heterogeneity (*I*^2^ = 69%, *p* = 0.01). There was no significant difference in the immediate reaction rates within 30 min post-vaccination between the Vi-DT group and the Vi-TT group, with a combined risk ratio of 0.48 [95% CI: 0.10, 2.40] and no observed heterogeneity (*I*^2^ = 37%, *p* = 0.21) (Figure 3).

In comparison to the control group, the Vi-DT group also did not show a statistically significant difference, with a combined risk ratio of 1.59 [95% CI: 0.14, 17.65], and moderate heterogeneity was observed (*I*^2^ = 63%, *p* = 0.07) (Figure 3).

##### 3.3.1.2 Solicited adverse events within 4 weeks after vaccination

**Figure 4:**
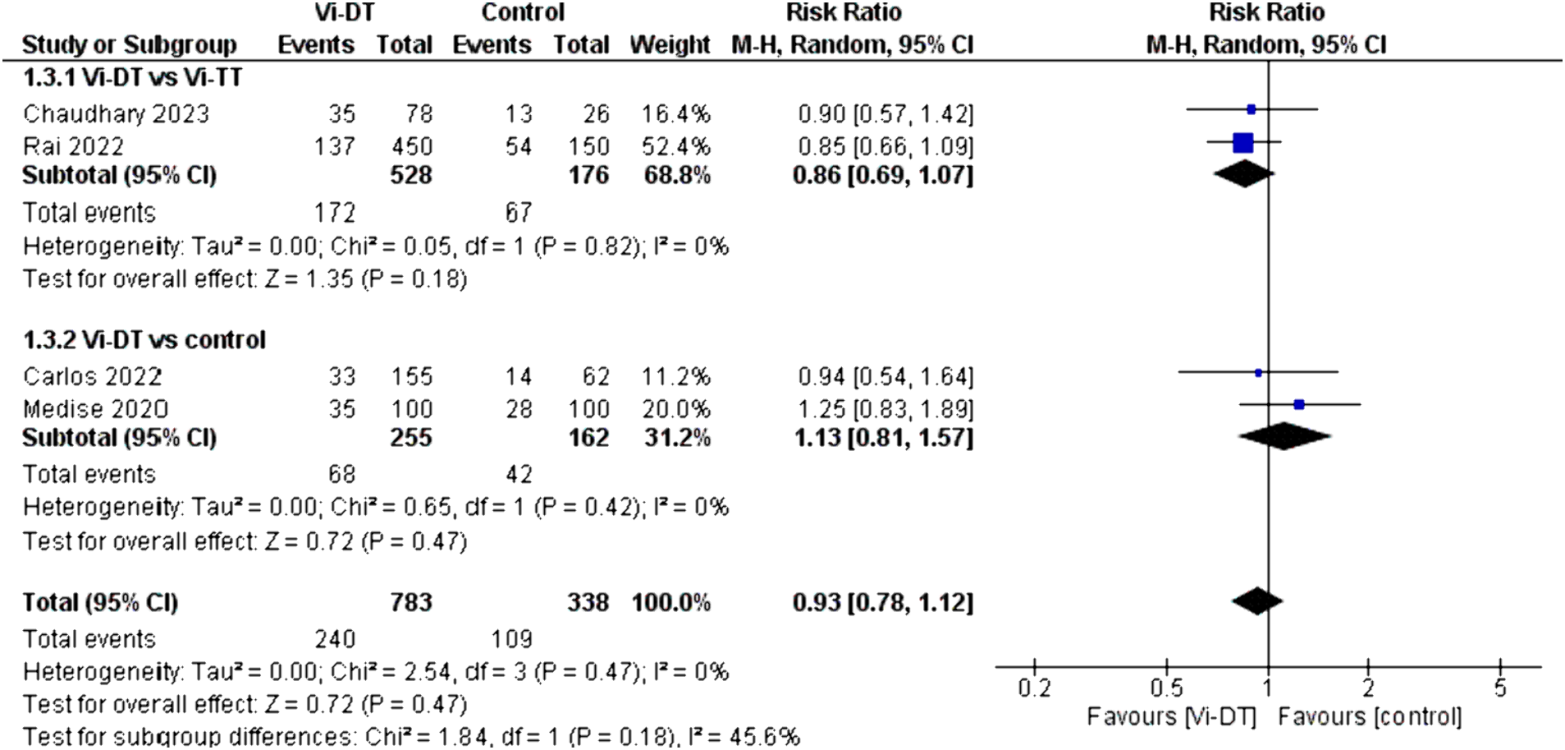
Solicited adverse events within 4 weeks after vaccination.

For solicited adverse events within 4 weeks after vaccination (Table 2), the combined risk ratio for Vi-DT versus control was 0.93 [95% CI: 0.78, 1.12] (Figure 4), indicating no significant increase in adverse events for those receiving Vi-DT compared to control. The heterogeneity for this outcome was low (*I*^2^ = 0%, *p* = 0.47) (Figure 4).

**Table 2:** Adverse events associated with typhoid vaccines.

Author	Year	Adverse events
Capeding et al.	2020	Initial reactions: following the initial dosage, one person experienced minor erythema, fever, and hypersensitivity, which all went away without any aftereffects. Another participant experienced pain/tenderness, fever, and diarrhea. Within 7 days after the initial dosage, there were 25.9% adverse events, compared to 19.3% in the comparator group. Adverse events in Group A included a febrile convulsion that occurred 4 weeks after the initial dosage. Within 4 weeks after the second dosage, Group B experienced an episode of gastroenteritis, while Group C experienced a febrile convulsion. Adverse occurrences over the first 4 weeks following the first dosage: 61.4% versus 68.4% in the comparator group. Within 4 to 24 weeks after the initial dosage, 4 incidences of pneumonia were among the adverse effects. There were three gastroenteritis episodes, two cases of febrile convulsions, and one frontal abscess case. It was determined that none of the SAEs were connected to the IP. Adverse events occurred in 11.4% of cases within 7 days after the second dose, compared to 9.1% in the comparator group. Adverse events within 4 weeks following the second dosage were 32.7%, whereas the comparative group experienced 27.3%.
Carlos et al.	2022	In all groups, the most frequent local immediate reaction seen was 13 (1.73%) participants experiencing discomfort or tenderness at the injection site. One participant in the control group (0.33%) and five (0.67%) in the MD Vi-DT developed erythema or redness at the injection site. In the MD Vi-DT group, three individuals (0.40%) had edema or induration. Fever was reported by one person (0.33%) in the control group, and headaches were reported by one participant (0.17%) in the MD Vi-DT group. In the MD Vi-DT group, there was only one recorded case of severe acute reaction (erythema/redness at injection site) for age stratum 3. Adverse events that occurred within 7 days of the vaccination: pain/tenderness at the injection site was the most common local AE reported with mild to moderate severity, occurring in 89 (8.93%) of the MD Vi-DT group, 88 (8.80%) of the SD Vi-DT group, and 110 (11.0%) of the control group. Severe AEs, which included swelling or induration and arthralgia, were reported in 10 participants (13.1%). In all groups, all serious solicited AEs were resolved in 1 to 3 days. The most frequent illness was mild to moderate upper respiratory tract infection, which affected 29 (3.87%) of the MD Vi-DT group, 38 (5.07%) of the SD Vi-DT group, and 10 (3.33%) of the control group. Severe fever cases were identified in one instance in each of the SD Vi-DT and control groups. AEs within 24 weeks after an 11-month-old child’s pneumonia illness with probable COVID-19. A case of dengue fever with probable COVID-19 in a toddler aged 1 year and 3 months, a seizure problem in a toddler aged 1 year and 4 months, and an early birth at 34 weeks of gestation were also recorded. One adult male patient, aged 31 years and 4 months, passed away from pulmonary tuberculosis and pneumonia. All were judged unrelated to vaccine.
Chaudhary et al.	2023	Within 30 min of the vaccination, the most common adverse event (AE) was headache, which occurred in 11 (3.67%) of the Vi-DT vaccine group 10 (3.33%) of which were local reactions and 1 (0.33%) of which was a systemic reaction; in contrast, 3 (3.00%) of the Vi-TT vaccine group were local reactions. Within 7 days following the vaccination, mild to moderate fever, headache, vomiting, and diarrhea were the most common adverse events (AEs). Of the participants in the Vi-DT group, 101 (33.67%) reported local AEs, while 41 (13.67%) reported systemic events. Of the participants in the Vi-TT group, 44 (44.00%) reported systemic AEs, and 37 (37.00%) reported local AEs. Within 4 weeks, AEs included mild to moderate cases of diarrhea, vomiting, fever, coughing, and nasopharyngitis in the Vi-DT group (18/100; 18.00%) and 63/300 (21.00%) of the Vi-TT group. All were determined to be unrelated to the vaccine and to have resolved amicably. Three serious adverse events (SAEs) were reported in the first 24 weeks: one (0.33%) in the Vi-DT vaccination group and two (2.00%) in the Vi-TT group. They included one medically assisted pregnancy termination (0.33%) in the Vi-DT group, one medically assisted pregnancy termination (1.00%) in the Vi-TT group, and one tubercular pleural effusion (1.00%) in the Vi-TT group. According to the site investigators and the data safety monitoring board, none of them had anything to do with the study films.
Medise et al.	2020	In the Vi-DT group, there were 6 (6%) and 1 (1%) systemic occurrence, whereas the immediate local reaction was 9 (9%) and 1 (1%). AEs within 31 min to 24 h: in the Vi-DT group, there were 8 (8%), 0 (0%), and 22 (22%) systemic events, and 14 (14%), local reactions, in the corresponding groups. AEs within 24–48 h: in the Vi-DT group, there were 1 (1%) and 0 (0%) local reactions, and 1 (1%) systemic event. Only 3 (3%), 1 (1%), and AEs within 48–72 h were systemic events in the Vi-DT group and Control group, respectively. Only systemic events within 72 h to 7 days caused AEs in the Vi-DT group (12%) and Control group (16%), respectively. The Vi-DT group experienced 35 (35%) and the Control group 28 (28%) AEs within 8–28 days, respectively.
Rai et al.	2022	Within 30 min after immunization, mild to moderate discomfort and tenderness at the injection site were noted in 14 participants in the Vi-DT vaccine group and 3 in the Vi-TT vaccine group. These adverse events (AEs) occurred in 16 (1.2%) and 4 (0.9%) of the vaccine groups, respectively. Erythema, or redness, was experienced by four participants—three from the Vi-DT vaccine group and one from the Vi-TT vaccine group. Following the Vi-DT vaccination, one participant complained of a headache, while another had nausea. AEs within 7 days of immunization; 260 (19.3%) Vi-DT vaccine groups and 115 (25.6%) Vi-TT vaccine groups experienced mild to moderate fever, headache, vomiting, and diarrhea, while seven Vi-DT vaccine groups and three Vi-TT vaccine groups experienced severe degree reactions. Within 4 weeks of immunization, there were 361 cases of diarrhea, vomiting, pyrexia, nasopharyngitis, and cough in the Vi-DT vaccine group and 143 cases in the Vi-TT vaccine group of adverse events (AEs). None of the mild to moderately severe side effects were linked to the study vaccine and all of them disappeared without a trace. Within a 24-week period, seven individuals reported experiencing serious adverse events (AEs). These included rotavirus gastroenteritis, pneumonia, pleural effusion, voluntary surgical or medical procedures, and cardiorespiratory arrest in three (0.7%) of the Vi-TT vaccine group and in (0.30%) of the Vi-DT vaccine group. Within 28 days of immunization, two women experienced abortions as major adverse events (AEs). Two participants each experienced acute viral gastroenteritis with mild dehydration, acute rotavirus gastroenteritis, lower lobe pneumonia, cardiopulmonary arrest, and right pleural effusion. The data safety monitoring board concurred that none of these severe adverse events could be linked to the trial vaccinations, as determined by the site investigators.

IP, investigation product; SAEs, serious adverse events; MD, multiple doses; SD, single dose; Vi-DT, diphtheria toxoid conjugated Vi-polysaccharide vaccine; TB, tuberculosis; Vi-TT, Vi-tetanus toxoid conjugated vaccine.

The analysis for solicited adverse events within 4 weeks after vaccination indicates a non-significant difference between the Vi-DT and Vi-TT groups, with a combined risk ratio of 0.86 [95% CI: 0.69, 1.07], and no heterogeneity (*I*^2^ = 0%, *p* = 0.82) (Figure 4).

When comparing Vi-DT with the control group, the combined risk ratio was 1.13 [95% CI: 0.81, 1.57], with no heterogeneity detected (*I*^2^ = 0%, *p* = 0.42) (Figure 4).

##### 3.3.1.3 Solicited adverse events within 7 days post-vaccination

**Figure 5:**
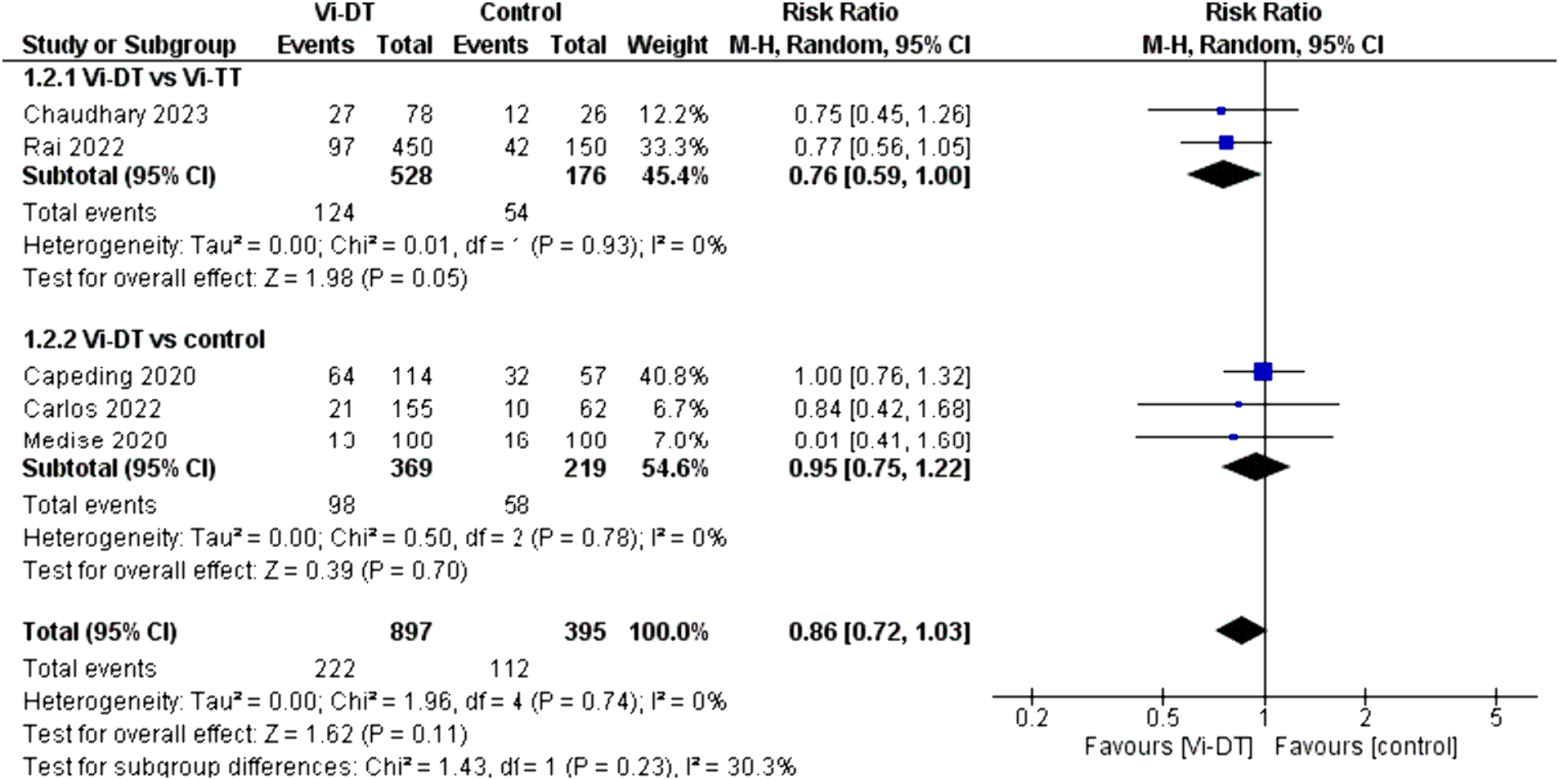
Solicited adverse events within 7 days post-vaccination.

The combined data for solicited adverse events within 7 days post-vaccination showed a risk ratio of 0.86 [95% CI: 0.72, 1.03] for the Vi-DT group compared to control, which suggests a non-significant trend toward fewer events in the Vi-DT group, with no detected heterogeneity (*I*^2^ = 0%) (Figure 5). The risk of solicited adverse events within 7 days post-vaccination did not significantly differ between the Vi-DT and Vi-TT groups, with a combined risk ratio of 0.76 [95% CI: 0.59, 1.00] (Figure 5), and no heterogeneity (*I*^2^ = 0%, *p* = 0.93). Against the control group, the Vi-DT group’s combined risk ratio was 0.95 [95% CI: 0.75, 1.22], and again, no heterogeneity was present (*I*^2^ = 0%, *p* = 0.78) (Figure 5).

#### 3.3.2 Efficacy outcome

##### 3.3.2.1 4-week seroconversion rate

**Figure 6:**
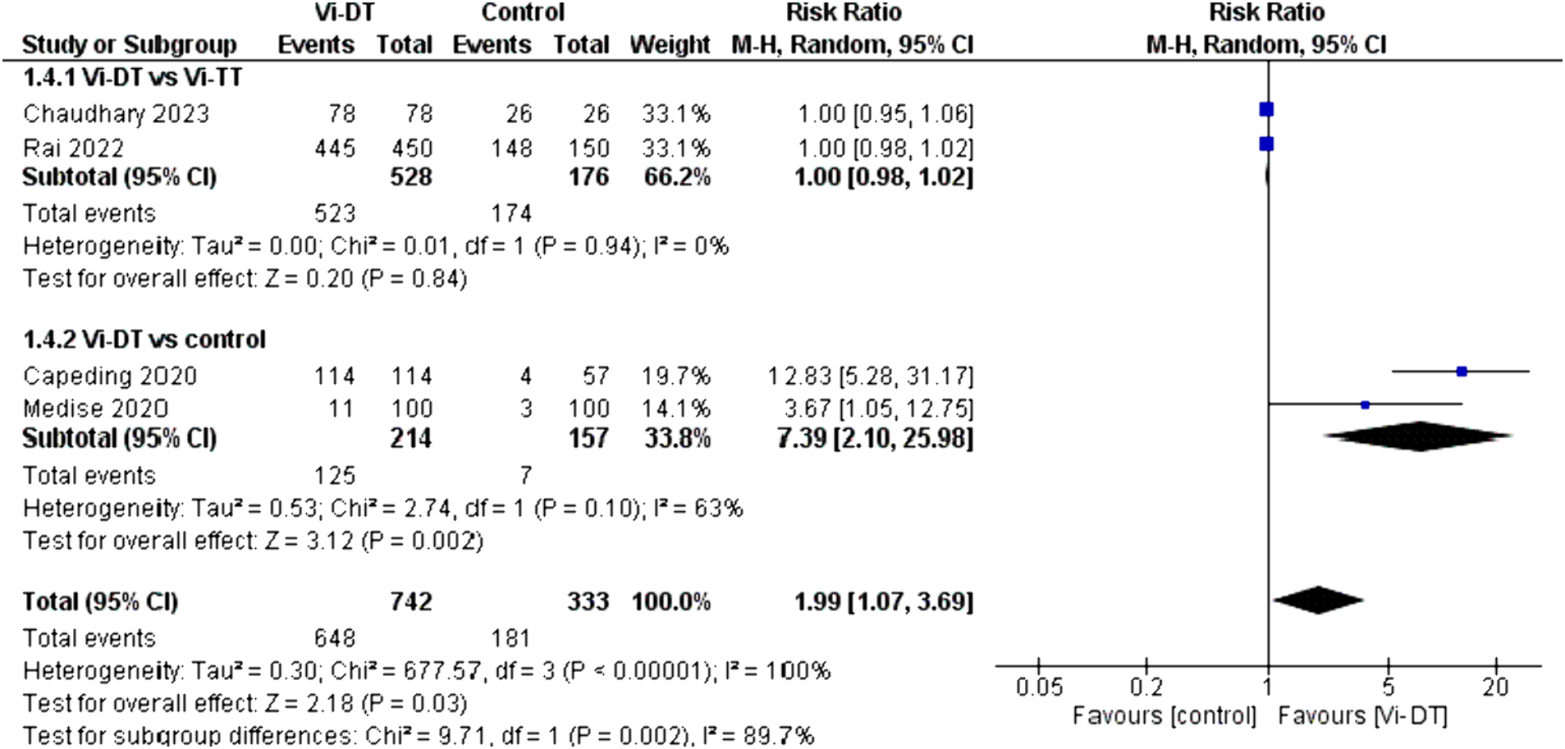
Four-week seroconversion rate.

The overall seroconversion rate at 4 weeks post-vaccination in the Vi-DT group compared to control showed a significantly higher rate, with a combined risk ratio of 1.99 [95% CI: 1.07, 3.69]. This statistically significant outcome indicates that Vi-DT is associated with a higher seroconversion rate. However, this finding is associated with a high level of heterogeneity (I^2^ = 100%), suggesting considerable variation in seroconversion rates across different control groups (Figure 6).

No significant difference was found between Vi-DT and Vi-TT, with a combined risk ratio of 1.00 [95% CI: 0.98, 1.02], and no heterogeneity was observed (*I*^2^ = 0%). The subgroup analysis comparing Vi-DT to the control group revealed a significant difference, with a combined risk ratio of 7.39 [95% CI: 2.10, 25.98] and substantial heterogeneity (*I*^2^ = 63%) (Figure 6).

#### 3.3.3 Adverse events associated with typhoid conjugate vaccines

The five studies included reported adverse events associated with typhoid conjugate vaccines. Most studies reported non-serious adverse events as immediate reactions, commonly fever, injection site pain/tenderness and redness. In contrast, adverse events within 7 days to 4 weeks post-vaccination were reported as mild to moderate fever, headache, vomiting, diarrhea, cough, and nasopharyngitis.

Serious adverse events reported within a 24-week timeframe included conditions such as pneumonia and gastroenteritis, rotavirus gastroenteritis, instances of voluntary surgical or medical procedures, pleural effusion, and cardiorespiratory arrest. The details of adverse events associated with TCV are presented in Table 2.

## 4 Discussion

Low- and middle-income countries (LMICs) are deeply concerned about preventing *S. typhi* infections due to the significant annual mortality rates and financial strain. Although vaccination is widely acknowledged as a highly cost-effective preventive strategy, our research is primarily geared toward examining the safety and immunogenicity of the innovative Vi-DT vaccine. This endeavor aims to improve the accessibility of the *S. typhi* vaccination. Our research targets explicitly children under the age of two, as they are especially vulnerable to the consequences of typhoid fever. Our comprehensive analysis, encompassing data from five Randomized Controlled Trials (RCTs), demonstrated the safety and effectiveness of the Vi-DT vaccine in children below the age of 2 years. The adverse events after Vi-DT were similar to those after control groups, with immediate reactions within 30 min postvaccination and solicited adverse events within 1 and 4 weeks post-vaccination. These were similar in the intervention and control groups. The findings support the use of this Vi-DT to expand typhoid vaccination in endemic regions, in line with WHO’s strategy.

A Phase II study on the Vi-DT vaccine developed by Bio Farma in Indonesia revealed its safety and efficacy in children aged 2–11 years old and adults up to 45 years old in the Philippines (Capeding et al., 2018). They administered two doses of the vaccine separated by 1 month. After the study above, Medise et al. (2020) researched the safety and efficacy of a single-dose vaccination in children aged 6 to under 24 months. Their findings revealed that 98.99% of the participants who received the Vi-DT vaccine exhibited seroconversion, compared to only 3.03% in the control group. Furthermore, 4 weeks following vaccination, anti-Vi IgG geometric mean titer (GMT) was significantly higher in the Vi-DT vaccine recipients compared to the control group. Additionally, the safety and immunogenicity of the Vi-DT vaccine (manufactured by SK Bioscience, Korea) were reported in clinical Phase I and II studies conducted on Filipino adults, followed by studies involving children and infants (Capeding et al., 2018, 2020). In the Philippines, a Phase II trial was conducted on children aged 6–23 months to evaluate the Vi-DT vaccine’s safety, immunogenicity, and reactogenicity compared to a placebo. Safety data was collected at three different time points: 60 min, 7 days, and 4 weeks after vaccination. The findings revealed no statistically significant disparities in the occurrence of adverse events between the cohort administered the trial vaccine and the placebo group. Predominantly, the adverse events documented were mild to moderate in severity. Nevertheless, it is significant to mention that one case of severe adverse event was recorded in the Vi-DT group, wherein a child experienced a febrile convulsion, which was ascribed to a urinary tract infection. The study’s authors have stated that this specific adverse event was not associated with the vaccination (Capeding et al., 2018). The study (Medise et al., 2020) noted that the test vaccine (Vi-DT) and the control vaccine led to pain as the most frequently reported immediate reaction. Interestingly, the incidence of pain was higher in the Vi-DT group. However, it’s essential to acknowledge that the study raised some concerns regarding deviations from the intended intervention. The primary study of the Phase III trial conducted in Nepal confirmed the safety and efficacy of the Vi-DT vaccine [34]. Similarly, in the Philippines, a Phase III trial including participants from 6 months to 45 years of age established the immunogenic equivalence and safety of both the multi-dose and single-dose forms of the Vi-DT vaccine (Franco-Paredes et al., 2016). These earlier findings reinforce our results concerning the safety and efficacy of Vi-DT and provide additional support for its consideration for WHO prequalification.

The idea behind conjugating the polysaccharide with a carrier protein is to address the shortcomings of using polysaccharides alone as immunogens and transform them into T cell-dependent antigens. These antigens are effective in children and infants and induce a booster response upon subsequent immunisations, leading to long-lasting protection. Using Diphtheria Toxoid (DT) as a carrier protein offers several advantages. Firstly, DT and ViPS are licensed and locally produced in many developing countries. Additionally, the anti-DT response generated by Vi-DT carries clinical benefits.

These benefits include an increased immune response and broader protection since the conjugation of Vi polysaccharide to the DT protein turns the vaccine into a T-cell dependent antigen, which is more immunogenic than T-cell independent polysaccharide vaccines (Berger, 1998). This increased immunogenicity is especially useful in children and infants, who usually have a lower response to polysaccharide antigens (Berger, 1998). Furthermore, the anti-DT response can provide extra protection against diphtheria, a deadly bacterial illness, in addition to the target disease protection afforded by the Vi polysaccharide components. Conjugate vaccines can be more cost-effective in the long term because they improve immunogenicity and duration of protection, lowering the need for frequent revaccinations and associated healthcare expenses. Finally, removing the necessity for a new carrier protein simplifies the licensing process for Typhoid Conjugate. Vaccines (TCV). These advantages add to the effectiveness and desirability of utilizing Vi-DT in immunization programs, particularly in resource-limited countries where diphtheria remains a hazard and typhoid fever is common.

### 4.1 Strengths and limitations

Our systematic review and meta-analysis represent the pioneering effort to examine the efficacy of the Vi-DT vaccine, specifically in children under the age of 2 years. The findings of this study are significant in advancing evidence-based decision-making for combating the current burden of Typhoid fever, especially in LMICs. Additionally, it provides supportive evidence for WHO prequalification of the Vi-DT vaccine. Notably, both WHO-prequalified TCVs are produced in India, and due to the high demand, there is a need for an increase in the supply. Vi-DT is the first Typhoid Conjugate Vaccine (TCV) to be manufactured outside of India, which will serve as a vital addition to the existing supply chain and enhance the diversity within the TCV portfolio.

When conducting a meta-analysis, we observed a significant heterogeneity in seroconversion results and immediate adverse event outcomes. The primary cause of this disparity is the different comparators utilized in the studies. We pooled the results of Vi-DT compared to a wide range of comparators, including placebo material with sodium chloride, IPV, MCV, and the authorized TCV, Vi-TT. Additionally, the studies included different forms of dosing, such as single and multiple doses. While this limitation lowers the certainty of the evidence, we addressed the source of heterogeneity using a leave-one-out test. By omitting the study (Medise et al., 2020) from the meta-analysis of immediate adverse events, the heterogeneity was resolved (*I*^2^ = 0), and the results were similar to those of the pooled results (RR = 0.44, 95% CI: [0.15, to 1.31]), which suggests no severe effect on our conclusion. However, it was not possible with the seroconversion outcome, as the leave-one-out test in multiple scenarios did not result in resolved heterogeneity.

To address the limitation of multiple comparators, we conducted a sensitivity analysis. The sensitivity analysis results showed that Vi-DT is comparable to Vi-TT in terms of seroconversion and adverse events. Furthermore, our analysis showed that Vi-DT is superior to placebo compared to SN and IPV. These sensitivity results suggest that Vi-DT is both immunogenic, safe, and not inferior to the established TCV, Vi-TT. It is important to note that one of the studies included in this report had some concerns about possible biased deviation from the intended intervention. However, when we omitted this study (Medise et al., 2020), it did not affect the meta-analysis results.

Moreover, the efficacy of the test vaccine was determined by the seroconversion of the bacterial antibodies. There is no clear antibody correlate of protection against *Salmonella* infection or clinical typhoid disease as many cases are asymptomatic, and it is argued that serum bactericidal antibody levels poorly correlate with disease (Jones et al., 2021). Longer follow-up for typhoid disease protection is still required. Also, as the included studies were conducted in endemic countries, individuals with subclinical typhoid may be included. However, it would be balanced between groups due to the randomization design of the included studies.

## 5 Conclusion

This review provides evidence supporting the safety and immunogenicity of the Vi-DT vaccine in children under the age of 2 years. The data indicates that Vi-DT does not elevate the risk of immediate reactions or solicited adverse events within 7 days or 4 weeks following vaccination compared to a control group or Vi-TT. Additionally, Vi-DT exhibits higher seroconversion rates at 4 weeks relative to the control and demonstrates immunogenicity that is not inferior to Vi-TT. These results support the potential use of Vi-DT as an effective typhoid fever vaccine for young children. Further studies are warranted to evaluate its long-term safety and effectiveness.

## Data availability statement

The original contributions presented in the study are included in the article/Supplementary material, further inquiries can be directed to the corresponding author.

## Author contributions

AMo: Conceptualization, Data curation, Formal analysis, Investigation, Methodology, Project administration, Resources, Software, Supervision, Validation, Visualization, Writing – original draft, Writing – review & editing. KA: Data curation, Formal analysis, Investigation, Methodology, Writing – original draft, Writing – review & editing. AMa: Investigation, Methodology, Writing – original draft, Writing – review & editing. ME: Data curation, Formal analysis, Investigation, Writing – original draft, Writing – review & editing. DN: Formal analysis, Investigation, Writing – original draft, Writing – review & editing. RA: Investigation, Methodology, Writing – original draft, Writing – review & editing. PP: Formal analysis, Investigation, Writing – original draft, Writing – review & editing. DB-A: Funding acquisition, Investigation, Methodology, Validation, Writing – original draft, Writing – review & editing. CL: Formal analysis, Investigation, Methodology, Writing – original draft, Writing – review & editing. AR-M: Data curation, Formal analysis, Funding acquisition, Investigation, Methodology, Project administration, Validation, Writing – original draft, Writing – review & editing.

## Footnotes

^1^ https://www.crd.york.ac.uk/prospero/display_record.php?RecordID=471203
