# Supplementary material for: Safety and immunogenicity of Vi-diphtheria toxoid typhoid conjugate vaccine among children below 2 years: a systematic review and meta-analysis
Source: Front Microbiol. 2024 Apr 5;15:1385834. doi: 10.3389/fmicb.2024.1385834 (PMC11026668; doi:10.3389/fmicb.2024.1385834)
Supplement: Supplementary file 1 [file Data_Sheet_1.PDF]

| Author | Year | Summarized Adverse Events |
|---|---|---|
| Capeding et al. | 2020 | 1. Immediate reactions post first dose: fever, pain/tenderness, diarrhea.<br>2. Within seven days post first dose: 25.9% adverse events.<br>3. Within four weeks post first dose: febrile convulsion. Post second dose: gastroenteritis and febrile convulsion.<br>4. 4-24 weeks post first dose: pneumonia, febrile convulsion, gastroenteritis, frontal abscess. Seven days post second dose: 11.4% adverse events. Four weeks post second dose: 32.7% adverse events. |
| Carlos et al. | 2022 | 1. Immediate reactions: pain/tenderness at injection site, erythema/redness, swelling/induration, fever, headache.<br>2. Seven days post-vaccination: most frequent was pain/tenderness. Four weeks post-vaccination: systemic events like fever, cough, and nasopharyngitis. |
| Rai et al. | 2022 | 1. Within 30 min post vaccination: pain, tenderness, erythema.<br>2. Seven days following vaccination: fever, headache, vomiting, diarrhea.<br>3. Within 4 weeks of vaccination: diarrhoea, vomiting, pyrexia, nasopharyngitis, cough.<br>4. Within 24-week: serious adverse events like pneumonia and gastroenteritis.<br>5. Within 28 days of vaccination: serious events like acute viral gastroenteritis, lower lobe pneumonia, cardiopulmonary arrest. |
| Chaudhary et al. | 2023 | 1. Within 30 mins postvaccination: pain, tenderness, redness at injection site, headache.<br>2. Within 7 days post-vaccination: fever, headache, vomiting, diarrhea.<br>3. Within 4 weeks: diarrhea, vomiting, fever, cough, nasopharyngitis. 24 weeks: serious adverse events including medical termination of pregnancy and tubercular pleural effusion. |
| Medise et al. | 2020 | 1. Immediate Reactions: Higher local (up to 9%) and systemic reactions (up to 22%) in the Vi-DT group compared to the control group within the first 24 hours.<br>2. 24-72 Hour Period: Reduced local reactions (1%) but steady systemic reactions (3-4%) in the Vi-DT group; minimal events in the control group.<br>3. 7-28 Days: Sustained systemic reactions in both groups, with 35% in Vi-DT and 28% in the control group. |
